# A New Setup for Simulating the Corrosion Behavior of Orthodontic Wires

**DOI:** 10.3390/ma14133758

**Published:** 2021-07-05

**Authors:** Polydefkis Papaioannou, Mona Sütel, Katrin Hüsker, Wolf-Dieter Müller, Theodosia Bartzela

**Affiliations:** 1 Department of Orthodontics, Dentofacial Orthopedics, and Pedodontics, Charité Center for Oral Health Sciences, Charité–Universitätsmedizin Berlin, Corporate Member of Freie Universität Berlin, Humboldt-Universität zu Berlin, and the Berlin Institute of Health, Assmannshauser Str. 4–6, 19147 Berlin, Germany; polydefkis.papaioannou@charite.de; 2 Department of Prosthodontics, Geriatric Dentistry and Craniomandibular Disorders, Charité Center for Oral Health Sciences, Dental Materials and Biomaterial Research, Charité – Universitätsmedizin Berlin, Corporate Member of Freie Universität Berlin, Humboldt-Universität zu Berlin, and the Berlin Institute of HealthAßmannshauser Str. 4–6, 14197 Berlin, Germany; mona.suetel@charite.de (M.S.); wolf-dieter.mueller@charite.de (W.-D.M.); 3 Department of Immunology, IMD Institute of Medical Diagnostics Berlin-Potsdam GbR, 12247 Berlin, Germany; k.hueske@imd-berlin.de

**Keywords:** orthodontic wires, corrosion, electrochemical test, ICP-MS analysis, Mini Cell System

## Abstract

The aim of this study was to create a new reliable setup to evaluate commercially available orthodontic wires used during orthodontic treatment. The setup includes various techniques applied for testing metal alloy materials. The materials were tested under extreme conditions to simulate their behavior in the mouth. The alloy composition of each wire was tested. The electrochemical (EC) testing and characterization of the corrosion performance of the wires was calculated by the electrochemical curves at pH = 1 in two different applied potentials to test the reaction of the material. The liquid collected after the EC measurements was analyzed by inductively coupled plasma-mass spectrometry (ICP-MS) to verify the reliability of the EC curves and for a more accurate evaluation of the corrosion behavior of the wires. Therefore, the EC measurements were compared to the actual values obtained from the released ions found in the solution. At the end, a surface analysis was performed to detect corrosion on the wires. In conclusion, this study developed a setup to test and better understand the corrosion behavior and ion release of the orthodontic wires, metal alloy dental materials, and other metals used in the oral cavity. This method can contribute to dental material selection in patients with underlying health conditions.

## 1. Introduction

The corrosion behavior of orthodontic wires and other dental materials containing metals or alloys has been studied both in vitro and in vivo using several different procedures, including surface analysis, electrochemical, and ion release measurements [1,2,3,4,5,6,7,8].

It is important to have a setup for predicting and checking the behavior of these materials because of the effect that different ions can have on the human body and health. For example, Ni and Cr ions, both released from orthodontic wires, are associated with hypersensitivity [9], cytotoxicity, and genotoxic effects [10,11]. There is evidence that Ni, Cr, and Co increase cancer risk in humans [12] and induce DNA damage in oral mucosa [13].

Ion release is influenced by external factors such as alterations in the oral environment, pH changes related to nutrition and oral microflora, temperature variations, influences by food and drink intake, and mechanical stress during mastication [7,14,15,16,17]. Electrochemical (EC) measurements were used to evaluate the ion release due to corrosion of different materials [18].

Furthermore, internal factors regarding the material itself, including its alloy composition, manufacture processing, and surface treatment, also play a role in the corrosion of the wires [19,20]. The manufacturing process of stainless steel (SS) orthodontic wires can lead to various forms of irregularities and an inhomogeneous surface as well as signs of crevice corrosion [20]. Moreover, the wire’s stress increases the corrosion rate [16]. 

Corrosion is not only related to biocompatibility but also to the appliance performance, as changes in the surface roughness of the wires could compromise orthodontic treatment [7]. The wires’ electrochemical characterization and stress corrosion can be carried out by the Mini Cell System (MCS) [1] through a set of four EC measurements. Due to the limitations of these measurements, inductively coupled plasma-mass spectrometer analysis can be used after every set of measurements to control the actual amount of ion release due to corrosion. Additionally, 3D digital microscopy at the end of each set of EC measurements can monitor the visibility of the corrosion products.

An alloy composition analysis can be performed using scanning electron microscopy and x-ray energy-dispersive spectroscopy analysis to evaluate substantial differences in alloy composition between the wires. Therefore, alloy composition analysis can be used to calculate the expected ion release obtained from the EC measurements.

Therefore, the aim of this study was to create an in vitro protocol that allows for the evaluation and a better understanding of the corrosion behavior of orthodontic wires and ion release in a fast and reliable way. This setup could theoretically be used to predict the ion release during a specific period of dental treatment with any of the selected materials, assisting the material selection, especially for patients with underlying chronic health conditions.

## 2. Methods and Materials

### 2.1. Experimental Measurements

The setup consisted of three different parts:

1. EDX surface analysis of the composition of the wire surface.
2. Assessment of corrosion stability with the use of a micro-electrochemical device and performance of corrosion simulation by polarization.
3. ICP-MS analysis of metal ion concentration in the test solution and inspection of the surface by 3D microscopy.

**Figure 1:**
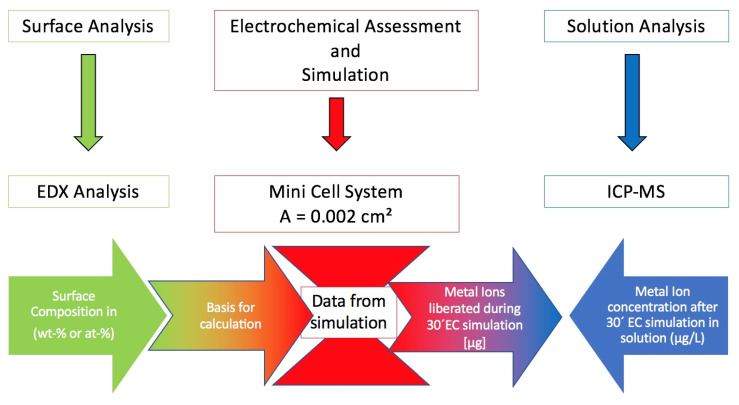
Summary of the setup. EDX, x-ray energy-dispersive spectroscopy; ICP-MS, inductively coupled plasma-mass spectrometer; EC, electrochemical.

A preview of the setup is shown in Figure 1.

### 2.2. Protocol and Materials

From the edge of each wire, a 2–3 cm long part was removed and used in the SEM/EDX alloy composition analysis.

**Figure 2:**
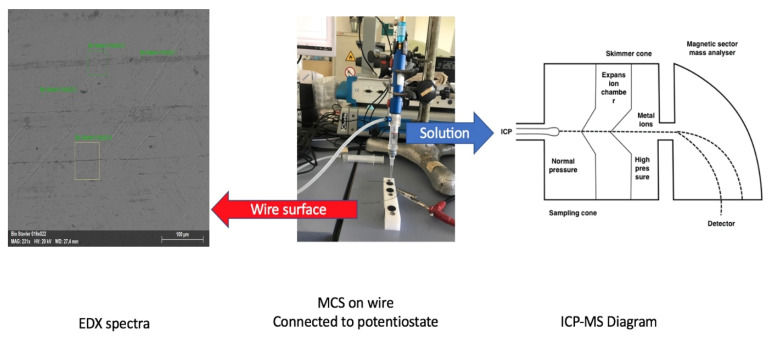
Techniques for assessing the in vitro behavior of orthodontic wires. MCS, Mini Cell System; EDX, x-ray energy-dispersive spectroscopy; ICP-MS, inductively coupled plasma-mass spectrometer.

The electrochemical characterization and simulation were realized using the Mini Cell System (MCS) [1], as shown in Figure 2. 

The system uses a three-electrode micro electrochemical cell, where a Pt wire is the counter electrode (CE), a saturated calomel electrode (SCE, 0.241V vs. NHE) is the reference electrode (RE), and the wire is the working electrode (WE). 

According to the protocol, after cleaning the surface of the wire with acetone, the tip of the MCS was placed on the surface of the wire. The contact area was 0.002 cm^2^.

The electrochemical test protocol begins with 10 min of open circuit potential (OCP) followed by cyclic voltammograms (CV) in the range between −0.6 and 1.0 V versus SCE with a scan rate of 10 mV/s. Five cycles were performed. At the end of each set of measurements, OCP was measured over 10 min (OCP-2). The last step was the simulation by polarization at 300 mV versus OCP or 750 mV versus OCP for 10 min. 

All measurements were performed with *n* = 3 for each wire type used in the experiment in Ringer’s solution at pH = 1 and room temperature (RT).

A preview of the protocol is provided in Figure 2.

The wires most commonly used in orthodontic treatment were selected. Three wires were composed of nickel–titanium (NiTi) alloy, and one was stainless steel (SS). Both the NiTi and SS wires were from FORESTADENT^®^ (Bernhard Foörster GmbH, Westliche Karl Friedrich Street 151, 75172 Pforzheim, Germany). All three dimensions of the NiTi wires used were of the same BioArchwire series. Three of each of the four types of wires were used for the experiment. A total of 72 sets of EC measurements were performed. On each wire, a total of six sets (each set was made up of OCP, CV, OCP-2, and a Potentiostatic measurement) of EC measurements were performed. An applied voltage of 300 mV versus OCP was used for the potentiostatic measurement of three selected areas. In three other areas, a voltage of 750 mV was tested. Table 1 shows the values of alloy composition provided by the supplier. 

### 2.3. Scanning Electron Microscopy and X-Ray Energy-Dispersive Spectroscopy Analysis

A 2–3 cm long part from the edge of each of the tested wires was cut and used for the SEM/EDX alloy composition analysis, where the atomic and weight percentages of each wire were obtained. At least three different areas were selected in each of the wires used to calculate the alloy composition.

The alloy composition was used in the calculation of the expected ion release through electrochemical polarization.

Therefore, a SEM (CamScanner Maxim, CamScan Electron Optics, Cambridge, UK) coupled with an EDX system XFlash (Quantax 6, Resolution 126 eV, Bruker, D, Berlin, Germany) was used. The analysis was performed at 20 keV at four different places on the wire surface. 

### 2.4. Electrochemical Measurements Using the Mini Cell System (MCS)

**Figure 3:**
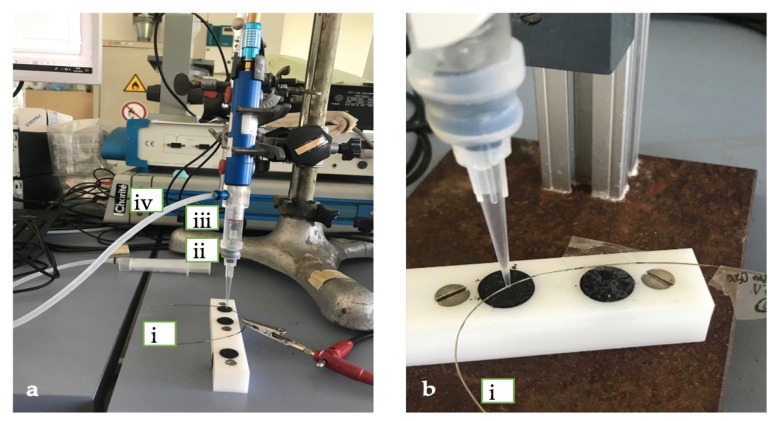
The setup for the electrochemical measurements (**a**) (**i**) Working electrode (orthodontic wire); (**ii**) Platinum wire (CE); (**iii**) Saturated calomel electrode (RE); (**iv**) Potentiostat and (**b**) a close-up picture of the contact point of the tip of the MCS with the working electrode (**i**) Working electrode (orthodontic wire).

The EC measurements were performed using a Mini Cell System. The MCS was attached to a Potentiostat SI 1286 Schlumberger and then to a computer. CorrWare software from Scribner Associates Inc., USA (Southern Pines, NC, USA) on Windows was used to run the measurements. The parts of the MCS are shown in Figure 3.

The EC simulations were performed on six different areas of each of the orthodontic wires: three for the polarization at 300 mV and three at 750 mV versus OCP. 

At the beginning of the set of measurements, an OCP was performed for 10 min until the surface reached a steady state.

Following OCP, a total of five cycles of CV were carried out. Thereafter, the surface was tested by applying a voltage between −0.6 mV and 1 mV in anodic and cathodic directions. The current produced on the surface was measured. 

When voltage runs in an anodic direction, corrosion takes place on the surface of the wire. Metal ions and electrons are produced due to oxidation. Metal ions are dissolved in the solution while the electrons give rise to a negative charge on the surface of the wire [8]. The negative charge on the surface positively attracts charged particles from the solution, and the metal ions produce a passive layer.

When voltage runs in a cathodic direction, reduction occurs, leading to the destruction of the passive protective layer. The electrons can react with the hydrogen ions found in the acidic electrolyte, water, and oxygen. The hydroxide ions formed can react with metal ions. These reactions can produce hydrogen gas and hydroxide products [8]. Appendix A shows the chemical reactions that occur during the CV measurement.

The interpretation of the logI versus E curves provides information on the wire’s corrosion behavior and aging process [8].

Every CV scan is divided into active, passive, and trans-passive areas. The active area is where the curve changes from an anodic to a cathodic direction. In this area, voluntary reactions occur, and zero current potential (E_0_) is present [8].

CorrView software from Scribner Associates Inc., USA (Southern Pines, NC, USA) was used for the analysis. 

**Figure 4:**
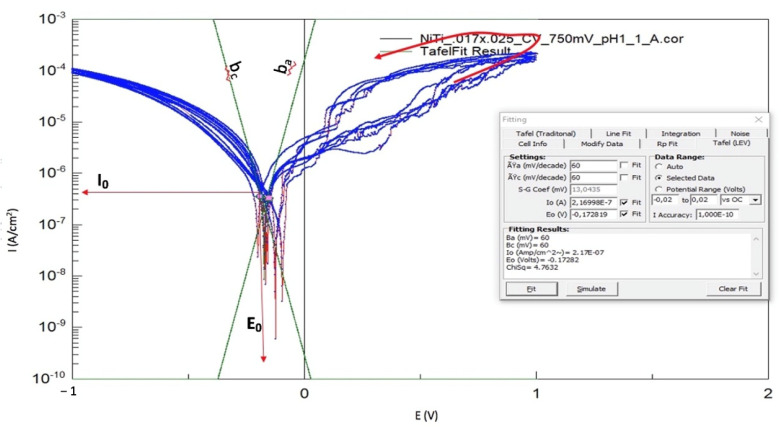
A cyclic voltammogram Log(i) versus E curves for NiTi wire showing the analysis window of CorrView 3.0 to estimate polarization resistance (Rp), corrosion current density (icorr) (corrosion current density (I_0_) values are marked in red), and corrosion potential (E_0_).

The results of each CV measurement were separated into five anodic scans. In the active area, two points were selected with a potential range of ±20 mV from E_0_. The Stern Geary procedure was performed [8,19]. The two points are marked in pink color in Figure 4. Corrosion current density (I_0_) can be determined by extrapolation of semi-logarithmic Evans’ diagram for the anodic (b_a_) and cathodic (b_c_) half-reactions as shown in Figure 4 [8,18,19].

The current density and corrosion rate can be calculated using Corrview, linear polarization resistance exchange, ‘’Tafel LEV’’ and ‘’Rp–Fit’’, and the measurement of the surface area, density, and equivalent weight of the wire. An example of how these values are calculated using Corrview is shown in Supplementary Materials A.

Equations (1)–(6) are used to calculate the electrochemically oxidized metal atoms of the wire surfaces.

(1) $$m=\sum^{\;}x_{i}m_{i}\;\;$$

(2) $$\rho =\sum^{\;}x_{i}\rho_{i}\;$$

(3) i∗t=z∗F∗n

(4) $$n=\sum^{\;}x_{i}\frac{m_{i}}{M_{i}}$$

(5) $$\frac{I*t}{F}*\frac{\sum^{\;}x_{i}M_{i}}{\sum^{\;}x_{i}z_{i}}$$

(6) I∗t=Q 

(*m_i_* is the mass of element in the alloy; ρ_I_ is the density of the element in the alloy; *I*/*i* is the current; t is the time; *z* is the number of transferred electrons; *F* is the Faraday constant; *M_i_* is the atom mass of element of the alloy; *Q* is the charge; * multiplication sign; and *n* is the amount of substance (mol)).

An example for calculating the density and equivalent mass of the wires is shown in Appendix B.

In addition, through potentiostatic measurements, the total charged pass (Q) could be calculated from i_corr_ versus the time curve using CorrView. An example of how Q is obtained using the curve from the potentiostatic measurements is shown in Supplementary Materials B.

**Figure 5:**
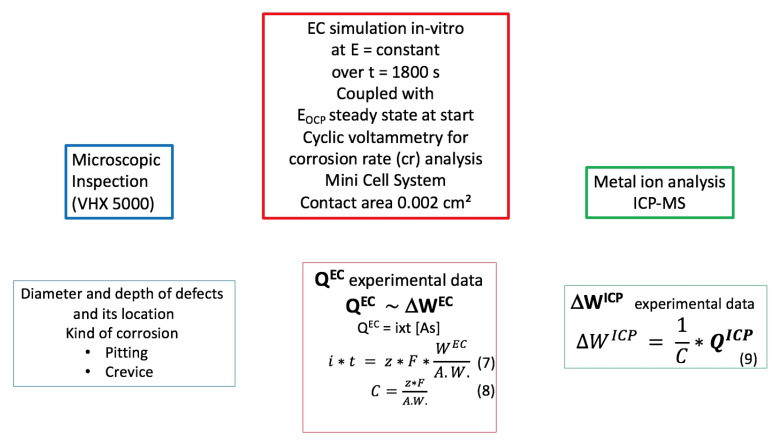
A schematic view of the protocol with an additional explanation of the formula of W.

The total charge Q (6) passed could then be combined with Faraday’s law (3), shown in Figure 5. Using the results obtained for the alloy composition in Equations (7) and (8), the expected mass of material removed from the wire at the two different potentials was calculated. The values obtained from each ion could then be compared with the values obtained from the solution analysis using Equation (9) [18].

(7) $$W=\frac{A.W.\;*\;Q}{zF}$$

- ***W*** = Mass of material removed (g);
- ***A.W.*** = Atomic weight of the sample (g/mol);
- ***Q*** = Total charge passed (i × time) (As);
- ***z*** = number of electrons transferred into the reaction;
- ***F*** = Faraday’s constant (As/mol).
- * = Multiplication sign

An example of how the mass of the material removed can be calculated using Equation (10) is shown in Appendix C.

From the mass of the material removed, the expected metal ion released in the 2 mL of the electrolyte was calculated. These values correspond to a measured area of 0.002 cm^2^. By measuring the whole surface of the wires, the expected ion release from the surface area of the entire wire can be estimated.

### 2.5. Inductively Coupled Plasma-Mass Spectrometry (ICP-MS) for Ion Analysis

At the end of each electrochemical measurement, the electrolyte was collected in a glass container. The MCS was rinsed three times with 2 mL of distilled water to collect all the remaining ions in the cell. The containers were sealed to avoid loss of electrolyte. The ion concentration analysis (μg/L) was performed at IMD Labor Berlin with an Icap Q ICP-MS (ThermoFisher Scientific, Waltham, MA, USA).

### 2.6. Assessment of Samples with 3D Microscopy

The digital microscope Keyence VHC 5000 was used at a magnification of 150 times to obtain 3D pictures of the areas on the wire where the electrochemical measurements were performed to verify their corrosion process.

### 2.7. Statistical Analysis

Analyses of variance (ANOVA) using Excel were used to assess the electrochemical data, ion concentrations analysis, and alloy composition results.

## 3. Results

### 3.1. Scanning Electron Microscopy and X-Ray Energy-Dispersive Spectroscopy Analysis (SEM/EDX)

The alloy composition measurements including the mean values of the atomic and weight percentages of each element of the four types of wires were calculated from the four areas tested on each wire.

**Figure 6:**
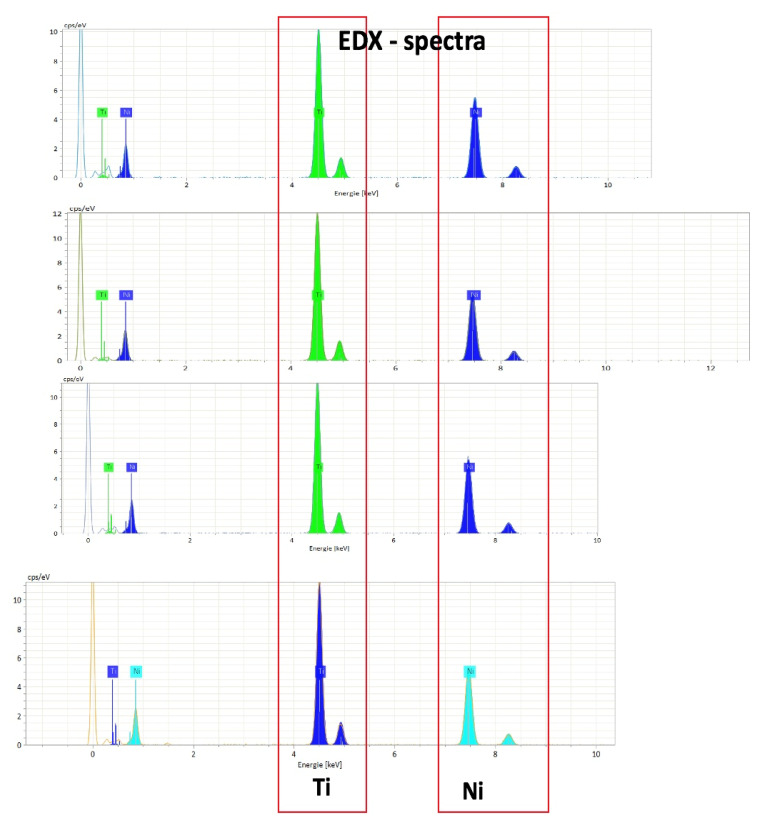
Results from the EDX analysis at four different areas at 20 keV, cps/eV vs. keV curves; EDX spectra of the four measurements.

Figure 6 presents an example of the results of the four areas of a NiTi 0.016″ × 0.022″ wire.

Table 2 shows the mean results from all wires divided into four categories according to their dimension and alloy composition.

### 3.2. Electrochemical Measurements Using a Mini Cell System (MCS)

A set of four EC measurements was applied to every measured area. First, the OCP measurement was carried out until a steady state was reached. Then, CV was applied, followed by a second OCP measurement before the potentiostatic measurement.

**Figure 7:**
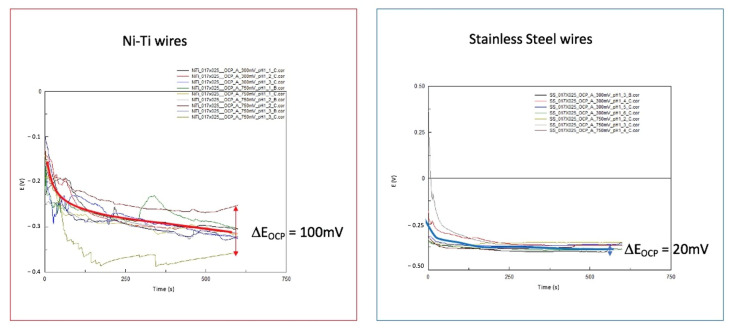
A collection of OCP E versus t curves from NiTi and SS wires at the beginning of EC measurements and simulations in Ringer’s solution at pH = 1, RT; the potential differences between the highest and lowest values are marked with red and blue arrows.

Figure 7 shows the OCP E versus time curves from a number of measuring areas of the SS and NiTi wires. In all cases, the OCP shifts in a cathodic direction over time. 

The OCP reached an endpoint in SS wires after 30 min at −0.37 ± 0.02 V versus SCE. 

The difference between the measurements narrows down over time. At the end of OCP1, the CV begins. This led to the assumption that before the CV, all wires had a similar condition.

In the case of NiTi wires, the general behavior was similar, with a cathodic shift in the OCP over time. At the end, the OCP was located at −0.300 ± 0.07 V versus SCE. The measurement variations increased over time compared to the results of the SS wires. In two areas of NiTi wires, the results showed distinct differences: one shifted in a more cathodic and the other in a more anodic direction.

**Figure 8:**
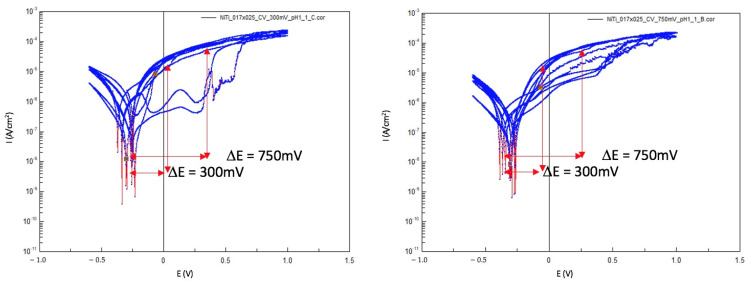
Two CV Log(i) versus E curves from two NiTi wires in Ringer’s solution pH = 1, RT; the positions for polarization are marked with red arrows.

**Figure 9:**
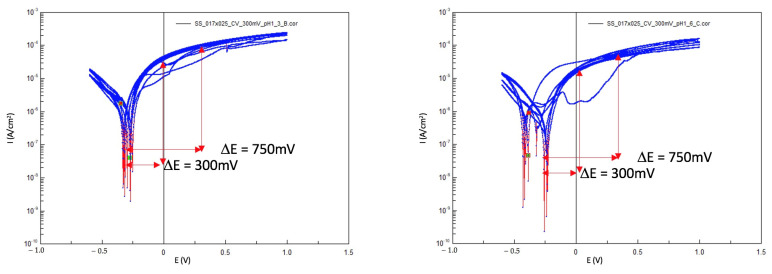
Two CV Log(i) versus E curves from two SS wires in Ringer’s solution pH = 1, RT; the positions for polarization are marked with red arrows.

Figure 8 and Figure 9 present examples of Log(i) versus E curves performed on NiTi and SS wires. Changes in the shape of the CV curves could be observed in both types of wires.

Characteristic passive behavior was present on the surface of the wire during the first two cycles of all cases. As the number of cycles increased, there was no signs of passivation, and the surface became active.

Another observation is that pitting corrosion was present on both the SS and NiTi wires.

Table 3 shows the electrochemical data collected from the different cycles. The focus of the study was the corrosion rate changes over time on each of the evaluated cycles.

The corrosion rate and potential were estimated using Corrview. The mean values for each of the four different wires from each of the five cycles are shown in Table 4 and Table 5.

**Figure 10:**
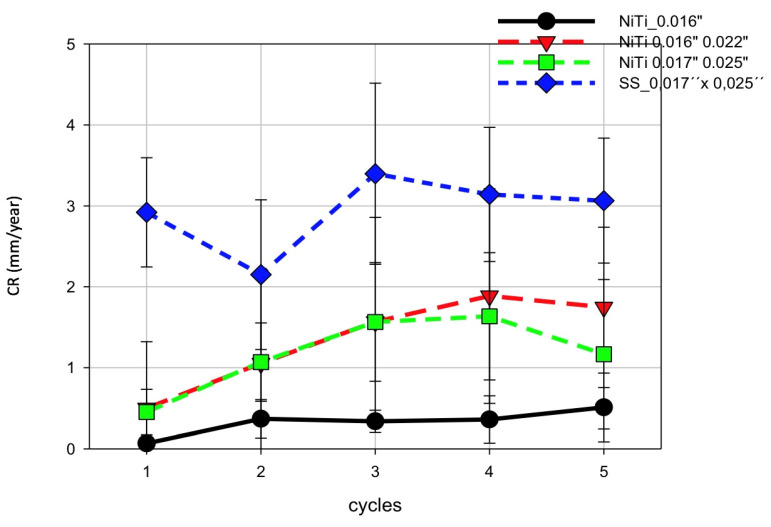
A comparison of CR of the investigated wires in Ringer’s solution at pH = 1 and RT.

A summary of the results of the corrosion rate in each cycle is presented in Figure 10.

**Figure 11:**
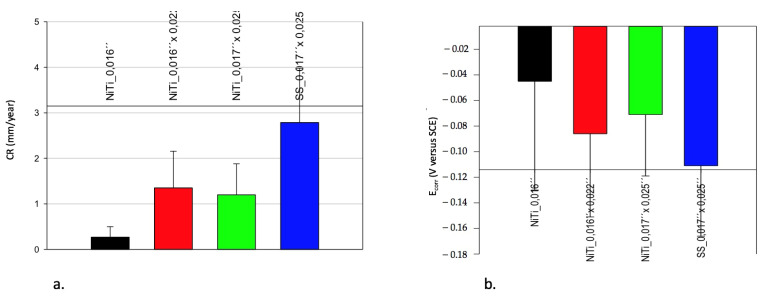
(**a**) Mean CR of wires being tested. (**b**) Mean E_corr_ of wires being tested in Ringer’s solution at pH = 1 and RT.

The CR on the SS wire surfaces was higher than on NiTi. Interestingly, there were differences between the various dimensions of the NiTi wires. In all cases, the CR increased along with the number of cycles and correlated with time since one cycle corresponded to a time interval of 340 s. The mean CR of SS was 3mm/year, that of NiTi 0.017″ × 0.025″ was 1.5 mm/year, and that of NiTi 0.016″ was 0.5. mm/year. Thus, NiTi 0.017″ × 0.025″ seems to have a CR three times higher than that of the 0.016″ NiTi wire. These results are also shown in Figure 11a,b, where the averaged CR and E_corr_ for the respective wires are summarized.

It was observed that the higher the negative value of E_corr_, the greater the CR of the wires.

**Figure 12:**
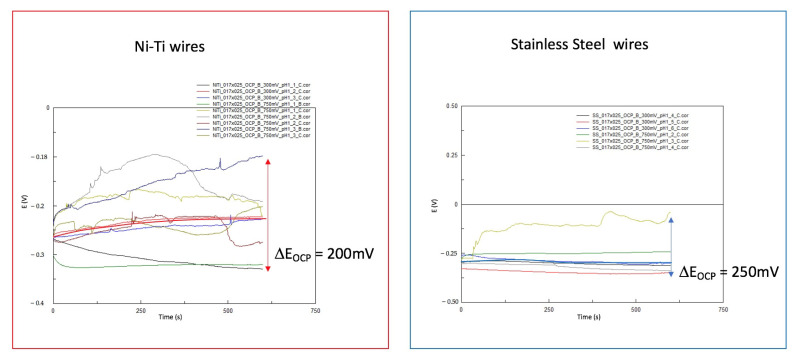
A collection of OCP E versus t curves of NiTi and SS wires after CV—aging of alloys—in Ringer’s solution at pH = 1, RT; the potential differences between the highest and lowest values are marked with red and blue arrows.

Following the CV obtained, the OCP-2 measurements were performed. Figure 12 shows examples of OCP versus time curves. In the SS wires, there were no significant changes observed over time. In the case of NiTi wires, considerable variations were observed.

**Figure 13:**
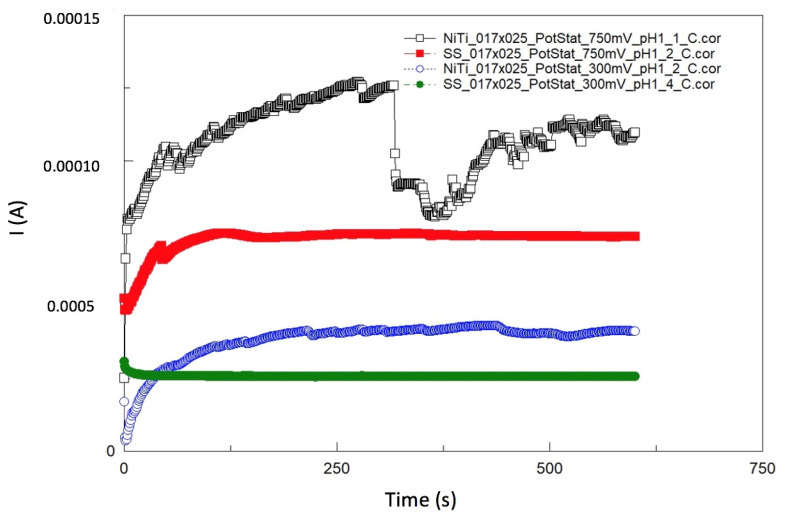
I vs. t curves of NiTi and SS wires during polarization with 300 mV and 750 mV versus OCP for simulation of oxidation in Ringer’s solution at pH = 1 (RT).

After OCP-2, the potentiostatic measurements were performed at 300 mV or 750 mV. Four selected curves are presented as examples in Figure 13. An oxidation reaction took place by applying a voltage of 300 mV or 750 mV anodically from the OCP. This reflects the conditions in the mouth when an anodic or cathodic load change occurs in a very short time in relation to the reduction and oxidation processes on the wire’s surface.

The transferred charges (Q) obtained from four representative curves of the NiTi_0.017″ × 0.025″ and SS_0.017″ × 0.025″ wires shown in Figure 13 are summarized in Table 6.

The calculations of ions released from Q were performed using Equation (10).

Based on the potentiostatic curves, the expected ion release was calculated at the two different applied potentials of the NiTi and SS wires. The results are shown in Table 7, Table 8 and Table 9.

### 3.3. Inductively Coupled Plasma-Mass Spectrometry (ICP-MS) for Ion Analysis

Table 10, Table 11 and Table 12 present the results of the ICP-MS analysis from the nickel–titanium and stainless-steel wires after measurements were made at the two different applied potentials.

In the case of NiTi wires, a considerable increase in the release of both Ni and Ti ions was observed when the potential was increased from 300 mV to 750 mV, and in certain cases by more than a factor of ten, as is shown in the results. The highest release was of Ni, followed by that of Ti, by about double or more.

In the case of the SS wires, the highest amount of ion release was found for Fe, followed by Cr, Ni, and Mn. However, the ion release was slightly lower at 750 mV versus OCP than at 300 mV versus OCP.

Table 13 presents an example of the results of W from ICP-MS for NiTi_ and SS_ 0.017″ × 0.025″ wires in μg/L and µg/2 mL. This helps the comparison of W with the results obtained from the EC measurements.

Table 14 presents an example of the total values of released ions from ICP-MS (µg/2 mL) in relationship to the total expected ions released (µg/2 mL) as obtained from the EC curves (shown in Table 7, Table 8 and Table 9) of the 0.017″ × 0.025″ SS and NiTi wires. 

### 3.4. Surface Analysis Using a 3D microscope

The morphology of the wires was assessed by making 3D images, from which it was observed that pitting and crevice corrosion, irregularities, some debris, scratches, and cracks could be seen. In general, all the wires had a smooth surface in non-tested areas. 

**Figure 14:**
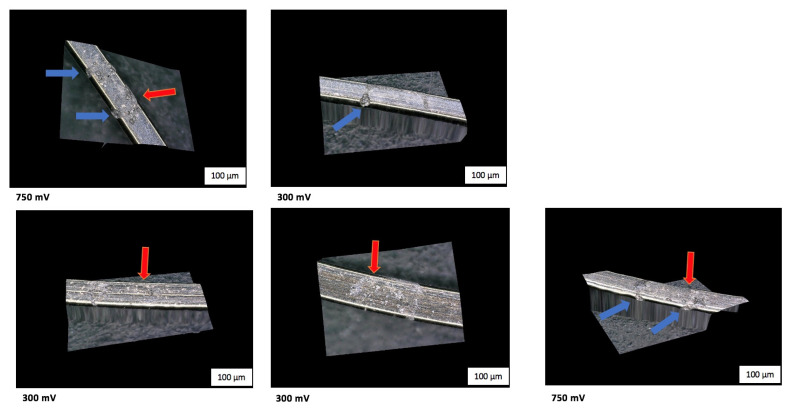
3D microscopic pictures of the Ni–Ti wire surfaces after polarization in Ringer’s solution pH = 1 at RT. The applied potential is provided underneath each image; red arrows show some examples of pitting corrosion; areas marked in blue show crevice corrosion.

**Figure 15:**
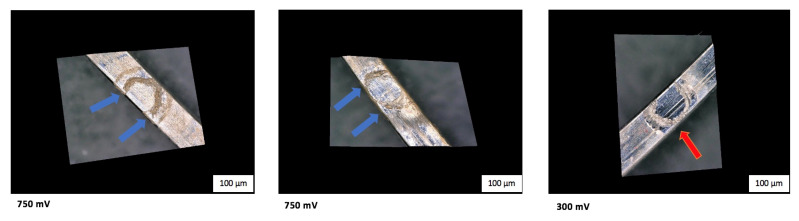
A number of 3D microscopic pictures of the SS wire surfaces after polarization in Ringer’s solution pH = 1 at RT. The applied potential is provided underneath each image; some areas of pitting corrosion are shown in red; some areas of crevice corrosion are shown in blue.

Every image shows a tested and a non-tested area. Irregularities on the non-tested surface of the wire that were present to a moderate degree were due to the manufacturing process. Some examples of the 3D pictures are shown in Figure 14 and Figure 15.

We observed the formation of rings (mostly on SS wires) made of cracks and pores as well as pitting and crevice formations or corrosion at the area where the walls of the tip of the MCS were in contact with the surface of the wire. This means that a higher amount of corrosion took place at these contact points. Some examples are presented in Figure 15.

## 4. Discussion

The aim of this study was to test and create a setup for the simulation of the corrosion of orthodontic wires for a more reliable prediction of their behavior.

First, we carried out an alloy composition analysis using scanning electron microscopy and x-ray energy-dispersive spectroscopy analysis (SEM/EDX). The results were used to calculate the corrosion rate and the expected released ion.

Electrochemical (EC) techniques were utilized through a Mini Cell System [1] to stress the in vivo simulation material and analyze the electrochemical performance. The corrosion rate and expected ion release can be calculated through the electrochemical curves, and the corrosion behavior can be characterized [8].

The test protocol was created to simulate an aging process, evaluate the corrosion rate, and provide information on the chemical stability of the dental metal alloys. Furthermore, it can identify pitting corrosion or even activation by different polarization states, which can be seen during cyclic polarization. Cyclic polarization is a procedure that has the potential to accelerate localized pitting or crevice corrosion, a process that would typically need a day or more to occur. 

The purpose of the chronoamperometry test (potentiostatic measurement) was to evaluate how the wire behaves anodically from the OCP at a potential of 300 mV or 750 mV. This process has been described in the ISO 10721 appendix for 300 mV vs. OCP.2. The higher potential was used only as a reference to evaluate whether higher currents charge the amount of ion release in the setting. 

One limitation of the protocol is that measurements on round wires like the NiTi_0.016″ wires were not as stable as those made on the rectangular-shaped wires. On the round wires was observed a higher loss of electrolyte, in the form of drops, compared to the rectangular-shaped ones. 

Additionally, crevice corrosion could lead to unpredictable ion release levels. Furthermore, due to the very small measurement area, crevice corrosion on the contact zone could lead to penetration of the electrolyte of the contact zone. This could give an explanation to the larger variability of results obtained from the SS wires, as SS seems to be more prone to crevice corrosion than NiTi, leading to different deformities and compromising the repeatability of the measurements.

Another limitation noticed was the formation of gas due to corrosion affecting the results of the EC measurements. This can be related to the small surface area where the measurements were performed.

Due to the limitations of electrochemical measurements, ICP-MS ion analysis tests and surface analyses were used as independent methods to control the reliability of the measurements and for a more accurate evaluation of the corrosion behavior of the wires [8]. Supplementary Material C shows how the results of corrosion rate, E_0_ from CV measurements and ICP-MS measurements were collected.

The working electrode was tested under OCP from electrochemical measurements to determine if the process reached a steady state. 

Apart from the state of the measuring surface, evaluation of OCP curves can provide information regarding the corrosion behavior of the material. An increasing OCP curve could represent the formation of a passive protective layer. In contrast, a decreasing OCP curve could indicate the development of a fragile porous hydroxide layer that may not protect the metal from further corrosion [19]. By comparing OCP with OCP-2, the slightly increasing curves of OCP-2 could mean the formation of a passive layer 

Via cyclic voltammetry, a voltage was applied in the anodic and cathodic directions of the wires to examine the wires’ corrosion and the aging process. Information on the stability of the oxide or hydroxide layers of the wire can be obtained from the shape of the CV curves as described in the Methods section. In addition, using the Corrview program and the corrosion potential, the corrosion rate and corrosion current density could be calculated. 

The results obtained from cyclic voltammetry showed that the expected average corrosion rate was generally higher for SS than for the NiTi wires. The expected ion release was also higher when obtained from the potentiostatic measurements of the SS wires compared to NiTi. This was also proved in the ICP-MS measurements.

One limitation of the protocol was the small surface area where the measurements were performed, leading to the inference that equilibrium was reached more quickly. 

Moreover, the signs of corrosion in the microscopic pictures of both wire types were mainly at the contact areas with the tip of the Mini Cell System. In contrast, homogeneous corrosion was expected throughout the measuring surface. 3D microscopic pictures of the Ni–Ti wire surfaces are shown in Figure 14. In Figure 15, the corrosion in SS wires is presented more clearly by the formation of rings on the surface. The higher signs of corrosion present on the SS wires correlate to the results of the EC measurements. 

The results of the five cycles can characterize the aging process of the wires. There was an increase in the corrosion rate from the first to the last cycle for both the SS and NiTi wires, showing that both types of wires are not stable. 

The ion release at 750 mV was higher by a factor of three compared to 300 mV of applied voltage by both the SS and NiTi wire-types, as shown in Table 7, Table 8 and Table 9.

The amount of the expected ion release of the NiTi wires according to the potentiostatic and the ICP-MS measurements, from 300 mV to 750 mV, were almost higher by a factor of ten with very high SD values at 750 mV.

The relationship between the ion release at 300 mV and 750 mV was not linear, and the reason for this was the passivity. Passivity occurs when an oxidation layer is formed on the layer that stops the anodic reaction and corrosion of the wire, leading to a decrease in the corrosion rate. The problem is that this layer is very fragile, leading to unpredictable localized forms of corrosion such as pitting and crevice corrosion, which were also observed in the microscopic images (Figure 14 and Figure 15). These unexpected forms of corrosion can compromise the use of the wires. At 300 mV, the creation of this oxide layer was possible. Still, at 750 mV, the solubility of the oxides was much higher, as the oxides were not stable enough, leading to a much higher release of ions, as observed during the ICP-MS analysis. As a result, Ni release at 750 mV, and, in some cases, the Ti release was ten times higher than that at 300 mV. 

In conclusion, these results were as expected, emphasizing the importance of the passivation layer of corrosion and that this experimental setup works. Furthermore, the fragility of the passive layer at such a highly applied potential of 750 mV can explain the high values of the SDs obtained at both potentiostatic and ICP-MS measurements compared to lower SD values on both tests at 300 mV.

In the case of the SS wires, the results of ion release from the ICP-MS measurements at 300 mV were slightly higher than those obtained with the application of 750 mV with very high SD values. The reason for this is the significant differences in the results obtained even on different areas on the same wire and the high corrosion rate of the SS wires found from the CV measurements. This leads to the inference that SS wires are not easily comparable, as they are not homogenous and may cause unpredictable ion release levels. Furthermore, a high number of irregularities in SS orthodontic wires has also been verified by other authors [20]. This can cause allergic reactions in the patients and lead to a metallic taste. In addition, Cr has been associated with hypersensitivity, cytotoxic, and genotoxic effects. Moreover, there is evidence that Cr increases the risk of cancer in humans [12]. 

Based on the results obtained from the ICP-MS measurements of the NiTi wires, the relationship between Ni and Ti can be observed. Even though the relationship of the alloy composition calculated from the SEM/EDX between Ni and Ti was at an approximate ratio of one to one, the release of Ni was almost double for 750 mV versus OCP than that of Ti. Therefore, nickel dissolves much faster than titanium. This can be a problem, as Ni may cause allergic reactions, cytotoxicity and induce DNA changes in oral mucosa [21]. Studies also show that Ni from fixed orthodontic appliances can be absorbed from the body [22].

As the data in Table 10, Table 11 and Table 12 show, the fluctuations in ion release are very high. Subsequently, metal ions can be released in the mouth in unexpected high amounts.

Further investigations are required to clarify the underlying reason for these fluctuations, besides the inhomogeneous surface of the wire due to the manufacturing process, especially in SS wires. The microscopic pictures confirm that pitting and crevice forms of corrosion occur in both alloys. Corrosion defects of different sizes can be observed, explaining the considerable differences in the amounts of ions released. Additionally, crevice corrosion, which is an accelerated form of corrosion, can be the reason. These forms of corrosion have been presented by other researchers [8].

These investigations show that it is necessary to study the stability of orthodontic wires using precise and better-resolving investigation techniques. The release of metal ions can cause impairment of the immune system and may lead to DNA changes and other side effects in the long-term.

In addition, through the microscopic images, higher signs of a crevice and pitting corrosion were observed on the contact point of the tip of the MCS with the wire. This was noticed in a higher degree on the SS wires by the formation of the ring-shaped signs of corrosion. An increasingly non-symmetric corrosion occurs at the contact areas compared to the whole measuring area, as was expected. Therefore, during orthodontic treatment, a higher corrosion rate is likely to appear at the areas where the metal wires are in contact with the brackets. Further studies are required to clarify this issue. 

## 5. Conclusions

This setup offers a method for evaluating the corrosion behavior of orthodontic wires. It also provides a better understanding of the behavior of the orthodontic wires.

Following the analysis of the NiTi and SS wires, the results of the increasing corrosion rate between the cycles of cyclic voltammetry show that the wires were not stable. 

The potentiostatic measurements for NiTi and SS wires showed a significant release of ion associated with the higher applied voltage, leading to increasingly unpredictable amounts of ion release. The same is expected to occur during the extreme conditions present in the mouth during treatment.

Further studies are needed to ascertain why the corrosion of orthodontic wires does not occur in a homogenous manner throughout the entire measurement area. A much higher corrosion degree was observed at the contact area of the walls of the MCS tip. This was shown by this in vitro setup, especially in the SS wires through the formation of ring corrosion signs.

The correlated changes in the ion release when changing the applied potential between the ICP-MS and the electrochemical measurements in the NiTi wires show that the setup can be used to evaluate this type of orthodontic wire. 

The SS wires tested were not comparable, leading to significant differences in ion release even on measurements performed on the same wires under the same conditions. The results obtained from the ICP-MS measurement provided a more accurate calculation of the corrosion behavior of the wires, showing unpredictable and high numbers of ion release. 

This protocol is a step for a better understanding and evaluation of the corrosion behavior of orthodontic wires. Further investigation is needed to evaluate the reason for this unpredictable ion release of the wires, especially in the case of the SS wires.

Further research is required to define the individual patient’s biological tolerance and selection criteria of orthodontic materials, especially in patients with underlying medical conditions. 

## Figures and Tables

**Table 1:** The amount of each metal present in the alloy as provided by the supplier.

Type	Dimensions	Amount (%)
NiTi-BioStarter^®^	0.016″	50.0–60.0 Ni, <0.5 Fe, <0.1 C, <0.1 Al, <0.1 O, <0.01 H, Rest Ti
NiTi-BioTorque^®^	0.016″ × 0.022″	50.0–60.0 Ni, <0.5 Fe, <0.1 C, <0.1 Al, <0.1 O, <0.01 H, Rest Ti
NiTi-BioFinisher^®^	0.017″ × 0.025″	50.0–60.0 Ni, <0.5 Fe, <0.1 C, <0.1 Al, <0.1 O, <0.01 H, Rest Ti
SS-Steel Archwire	0.017″ × 0.025″	≤0.12 C, ≤1.5 Si, ≤2.0 Mn, 16.0–18, 0 Cr, ≤0.8 Mo, 6.0–9.0 Ni, ≤0.045 P, ≤0.03 S, Rest Fe

**Table 2:** The alloy composition mean values of the four different types of wires.

Wire	Elements	Mean Atomic (%)	SD	Mean Weight (%)	SD
SS_0.017″ × 0.025″	Fe	71.147	0.587	71.858	0.589
	Cr	20.157	0.015	18.955	0.013
	Ni	8.041	0.043	8.537	0.047
	Mn	0.985	0.035	0.975	0.035
NiTi_0.016″	Ni	45.427	0.999	50.510	0.995
	Ti	54.573	0.999	49.497	1.003
NiTi_0.016″ × 0.022″	Ni	46.388	1.609	51.467	1.610
	Ti	53.613	1.611	48.535	1.611
NiTi_0.017″ × 0.025″	Ni	47.580	0.680	52.653	0.675
	Ti	52.420	0.680	47.347	0.675

**Table 3:** The results for the mean corrosion potential E_corr_ at each of the five cycles versus SCE.

Wires	Mean E_0_ (V vs. SCE)	SD of E_0_	Mean Corrosion Rate (mmPY)	SD of Corrosion Rate
NiTi_0.016″	−0.045	±0.085	0.268	±0.231
NiTi_0.016″ × 0.022″	−0.086	±0.065	1.50	±0.805
NiTi_0.017″ × 0.025″	−0.071	±0.048	1.198	±0.681
SS_0.017″ × 0.025″	−0.111	±0.055	2.786	±1.193

**Table 4:** The results for the corrosion rate (CR) at each of the five cycles.

Wires	CR Cycle 1 (mmPY)	CR Cycle 2 (mmPY)	CR Cycle 3 (mmPY)	CR Cycle 4 (mmPY)	CR Cycle 5 (mmPY)
NiTi_0.016″					
Mean (mmPY)	0.067	0.369	0.338	0.361	0.531
STDEV	±0.065	±0.237	0.137	0.291	0.424
NiTi_0.016″ × 0.022″					
Mean (mmPY)	0.499	1.053	1.567	1.886	1.746
STDEV	0.823	1.126	1.291	1.327	0.991
NiTi_0.017″ × 0.025″					
Mean (mmPY)	0.452	1.069	1.565	1.635	1.167
STDEV	0.281	0.484	0.733	0.786	0.923
SS_0.017″ × 0.025″					
Mean (mmPY)	2.920	2.150	3.397	3.142	3.065
SD	0.675	0.926	1.120	0.829	0.773

**Table 5:** The results for the mean corrosion potential in each of the five cycles.

Wires	Eo (V vs. SCE) Cycle 1	Eo (V vs. SCE) Cycle 2	Eo (V vs. SCE) Cycle 3	Eo (V vs. SCE) Cycle 4	Eo (V vs. SCE) Cycle 5
NiTi_0.016″					
Mean (mmPY)	−0.012	−0.040	−0.338	−0.039	−0.063
SD	±0.078	0.084	0.147	0.045	0.071
					
NiTi_0.016″ × 0.022″					
Mean (mmPY)	−0.043	−0.131	−0.091	−0.091	−0.094
SD	±0.074	0.142	0.084	0.081	0.053
NiTi_0.017″ × 0.025″					
Mean (mmPY)	−0.042	−0.092	−0.093	−0.078	−0.051
SD	±0.052	0.048	0.039	0.045	0.054
SS_0.017″ × 0.025″					
Mean (mmPY)	−0.111	−0.142	−0.101	−0.102	−0.098
SD	±0.003	0.077	0.020	0.036	0.023

**Table 6:** Summary of charges (Q) and the calculated mean metal ion mass from Figure 13.

Wires	E (mV Versus OCP)	Q (As)	Total W_EC_ (µg/2 mL)
NiTi_0.017″ × 0.025″	750	0.0641	11.9
NiTi_0.017″ × 0.025″	300	0.0228	4.23
SS_0.017″ × 0.025″	750	0.0436	8.28
SS_0.017″ × 0.025″	300	0.0154	2.92

**Table 7:** The average expected ion release from the NiTi wires at 300 mV.

Wires at 300 mV	Total Ti μg in 2 mL	SD	Total Ni μg in 2 mL	SD	Total Ions Released (µg/2 mL)	SD
NiTi_0.016″	0.786	0.284	0.643	0,241	1.500	0.707
NiTi_0.016″ × 0.022″	1.56	0.817	1.294	0.766	2.750	1.581
NiTi_0.017″ × 0.025″	1.304	0.819	1.196	0.762	2.500	1.581

**Table 8:** The average expected ion release from the NiTi wires at 750 mV.

Wires at 750 mV	Total Ti μg in 2 mL	SD	Total Ni μg in 2 mL	SD	Total Ions Released (µg/2 mL)	SD
NiTi_0.016″	6.035	6.875	6.035	6.142	13.000	13.015
NiTi_0.016″ × 0.022″	7.260	1.255	6.169	2.440	13.429	2.440
NiTi_0.017″ × 0.025″	6.782	1.727	6.196	1.548	13.000	3.240

**Table 9:** The average expected ion release from the SS wires at 300 mV and 750 mV.

Potential Applied on the SS Wires in mV	Total Fe μg in 2 mL	SD	Total Ni μg in 2 mL	SD	Total Cr μg in 2 mL	SD	Total Mn μg in 2 mL	SD	Total Ions Released μg in 2 mL	SD
**300**	2.750	1.426	0.309	0.080	0.762	0.407	0.040	0.028	3.867	2.036
**750**	9.914	2.160	0.999	0.454	2.771	0.626	0.146	0.032	13.857	3.132

**Table 10:** The average amount of Ti and Ni ions being measured when 300 mV was applied on the three different dimensions of the NiTi wires.

Wires at 300 mV	Average Amount of Titanium Ions in µg/L	SD	Average Amount of Nickel Ions in µg/L	SD
NiTi_0.016″	3.18	2.950	7.885	4.231
NiTi_0.016″ × 0.022″	60.193	19.455	95.407	25.808
NiTi_0.017″ × 0.025″	9.910	4.397	11.988	8.608

**Table 11:** The average amount of Ti and Ni ions being measured when 750 mV was applied on the three different dimensions of the NiTi wires.

Wires at 750 mV	Average Amount of Titanium Ions in µg/L	SD	Average Amount of Nickel Ions in µg/L	SD
NiTi_0.016″	9.140	5.298	13.822	13.451
NiTi_0.016″ × 0.022″	606.640	512.667	1084.487	926.670
NiTi_0.017″ × 0.025″	252.805	65.982	430.429	39.317

**Table 12:** The average amount of Fe, Ni, Cr, and Mn ions being measured when 300 mV or 750 mV was applied on the three different dimensions of the SS wires.

Applied Potential on the SS Wires in mV	Average Amount of Iron Ions in µg/L	SD	Average Amount of Nickel Ions in µg/L	SD	Average Amount of Chromium Ions in µg/L	SD	Average Amount of Manganese Ions in µg/L	SD
**300**	431.252	752.796	57.394	103.333	102.998	195.985	11.344	14.748
**750**	341.613	705.092	45.697	95.528	83.093	158.971	8.967	12.435

**Table 13:** A summary of the results of total W in μg/L and μg/2 mL from the ICP-MS analysis for the SS and NiTi wires with dimensions of 0.017″ × 0.025″.

Wires	E (mV Versus OCP)	Total W_ICP-MS_ (μg/L)	Total W_ICP-MS_ (µg/2 mL)
NiTi_0.016″	750	22.962	0.046
NiTi_0.016″	300	11.065	0.022
NiTi_0.016″ × 0.022″	750	1691.127	3.382
NiTi_0.016″ × 0.022″	300	155.600	0.311
NiTi_0.017″ × 0.025″	750	683.234	12360
NiTi_0.017″ × 0.025″	300	21.898	0.044
SS_0.017″ × 0.025″	750	476.57	0.959
SS_0.017″ × 0.025″	300	602.988	1.210

**Table 14:** The relation between the expected ion release from the potentiostatic measurements and the metal ions released as calculated from the ICP-MS analysis.

Wire	E (mV Versus OCP)	W_EC_/W_ICP_	W_ICP_/W_EC_ × 100 (%)
NiTi_0.016″	750	282.61	0.35
NiTi_0.016″	300	68.18	1.47
NiTi_0.016″ × 0.022″	750	3.97	25.18
NiTi_0.016″ × 0.022″	300	8.84	11.31
NiTi_0.017″ × 0.025″	750	9.56	10.46
NiTi_0.017″ × 0.025″	300	56.82	1.76
SS_0.017″ × 0.025″	750	14.45	6.92
SS_0.017″ × 0.025″	300	3.20	25.85

## Data Availability

The data presented in this study are available on request from the corresponding author. The data are not publicly available as raw/processed data required to reproduce these findings cannot be shared at this time as the data also forms part of an ongoing study.

## Supplementary Materials

The following are available online under Supplementary Material A, B, and C at https://www.mdpi.com/article/10.3390/ma14133758/s1, Figure S1: An example cell information that were used with Corroview to estimate polarization resistance (Rp), corrosion current density (icorr) (corrosion current density (I_0_); Figure S2: An example showing the two-point are marked on the curve, and the ‘’Tafel LEV’’ option is used to obtain Stern coefficient.; Figure S3: An example showing cell information marked with red arrows.; Figure S4: An example is showing the Corrosion rate, Polarization resistance (Rp), corrosion current density (I_0_), and corrosion potential (E_0_) calculation using ‘’RP – Fit’’; the results are marked with red arrows; Figure S5: An example showing the calculation of Q from I versus time curve using ‘’RP – Fit’’.; Table S1: showing results of corrosion rate, E_0_ from CV measurements and ICP-MS measurements from the NiTi_0.016″ × 0.022″

## Appendix A

“Oxidation:

1. Me -> Me^z+^ + ze^−^

Reduction:

2. Me^z+^ + ze -> Me
3. zH^+^ + ze^−^ -> zH_2_
4. zO_2_ + H_2_O + ze^−^ -> zOH^−^
5. zOH^−^ + Me^z+^ -> zMeOH”

[8]

## Appendix B

Density Calculated Using: −WT% of alloy (calculated by SEM/EDX measurement)

For a Nickel–Titanium Alloy

Ti: 4.51 g/cm^3^; Ni: 8.91 g/cm^3^

WT: 47.33% Ti and 52.67% Ni

0.47 × 4.50 + 0.53 × 8.91 = 6.8373 g/cm^3^

Equivalent Mass Calculation: AT% of alloy (calculated by SEM/EDX measurement)

Need the molecular weight (atom mass) of each element

Molecular weight Ti: 47.88 g/mol (*n* = 4 electrons) Ni: 58.69 g/mol (*n* = 2 electrons)

0.52(AT%) × 47.88/4 + 0.48 × 58.69/2 = 20.31 g

## Appendix C

Atomic %: 0.2 Cr, 0.72 Fe, 0.08 Ni (obtained from the alloy composition analysis)

*n*: 3 Cr, 2 Fe, 2 Ni;

Molecular weight: 51.996 g/mol Cr, 55.845 g/mol Fe, 58.693 g/mol Ni

A.W = 0.2 × 51.996 + 0.72 × 55.845 + 0.08 × 58.693 = 55,40162 g/mol.

Z = 0.2 × 3 + 0.72 × 2 + 0.08 × 2 = 2.2.

Q = 0.019636 (As) (obtained using CorrView)

W(m) = 0.019636 × 55.40162/2.2 × 96 485 (F) = 5.125 × 10^−6^ g in 2 mL.

Fe = 0.72 × 5.125 = 3.69 μg in 2 mL

Ni = 0.08 × 5.125 = 0.41 μg in 2 mL

Cr = 0.2 × 5.125 = 1.025 μg in 2 mL
