# Supplementary material for: A New Setup for Simulating the Corrosion Behavior of Orthodontic Wires
Source: Materials (Basel). 2021 Jul 5;14(13):3758. doi: 10.3390/ma14133758 (PMC8269849; doi:10.3390/ma14133758)
Supplement: Supplementary file 1 [file materials-14-03758-s001.zip › materials-1218331-supplementary.pdf]

materials

MDPI

Article

A New Setup for Simulating the Corrosion Behavior of Orthodontic Wires

Polydefkis Papaioannou [1,2], Mona Sütel [2], Katrin Hüsker [3], Wolf-Dieter Müller [2] and Theodosia Bartzela [1,*]

[1] Department of Orthodontics, Dentofacial Orthopedics, and Pedodontics, Charité – Universitätsmedizin Berlin, corporate member of Freie Universität Berlin, Humboldt-Universität zu Berlin, and the Berlin Institute of Health, CC3 Institute of Dental and Craniofacial Sciences, Assmannshauser Str. 4–6, 19147 Berlin, Germany; polydefkis.papaioannou@charite.de (P.P.); theodosia.bartzela@charite.de (T.B.)

[2] Department of Dental, Oral and Maxillary Medicine, Charité – Universitätsmedizin Berlin, corporate member of Freie Universität Berlin, Humboldt-Universität zu Berlin, and the Berlin Institute of Health, CC3 Institute of Dental and Craniofacial Sciences, Assmannshauser Str. 4–6, 19147 Berlin, Germany; polydefkis.papaioannou@charite.de; mona.suetel@charite.de (M.S.); wolf-dieter.mueller@charite.de (W.-D.M.)

[3] Department of Immunology, IMD Institute of Medical Diagnostics Berlin-Potsdam GbR, 12247 Berlin, Germany; k.hueske@imd-berlin.de

* Correspondence: theodosia.bartzela@charite.de

Supplementary Materials A:

CELL INFO

Surface Area measured from microscope.

Density calculated using:- WT % of alloy from EDX measurement.

An example with NiTi: Density of : Ti - 4.51 g/cm^3

Ni - 8.91 g/cm^3

WT: 47.33% Ti and 52.67% Ni

$0.47 \times 4.50 + 0.53 \times 8.91 = 6.8373\ g/cm^3$

Equivalent Mass Calculation: AT% from EDX used for equivalent mass calculation.

Molecular weight of Ti: 47.88 g/mol n = 4 electrons

Ni: 58.69 g/mol n = 2 electrons

$0.52 \times 47.88 / 4 + 0.48 \times 58.69 / 2 = 20.31$ g

Figure S1. An example of the cell information that were used with Corroview to estimate polarization resistance (Rp), corrosion current density (icorr) (corrosion current density (I_0).

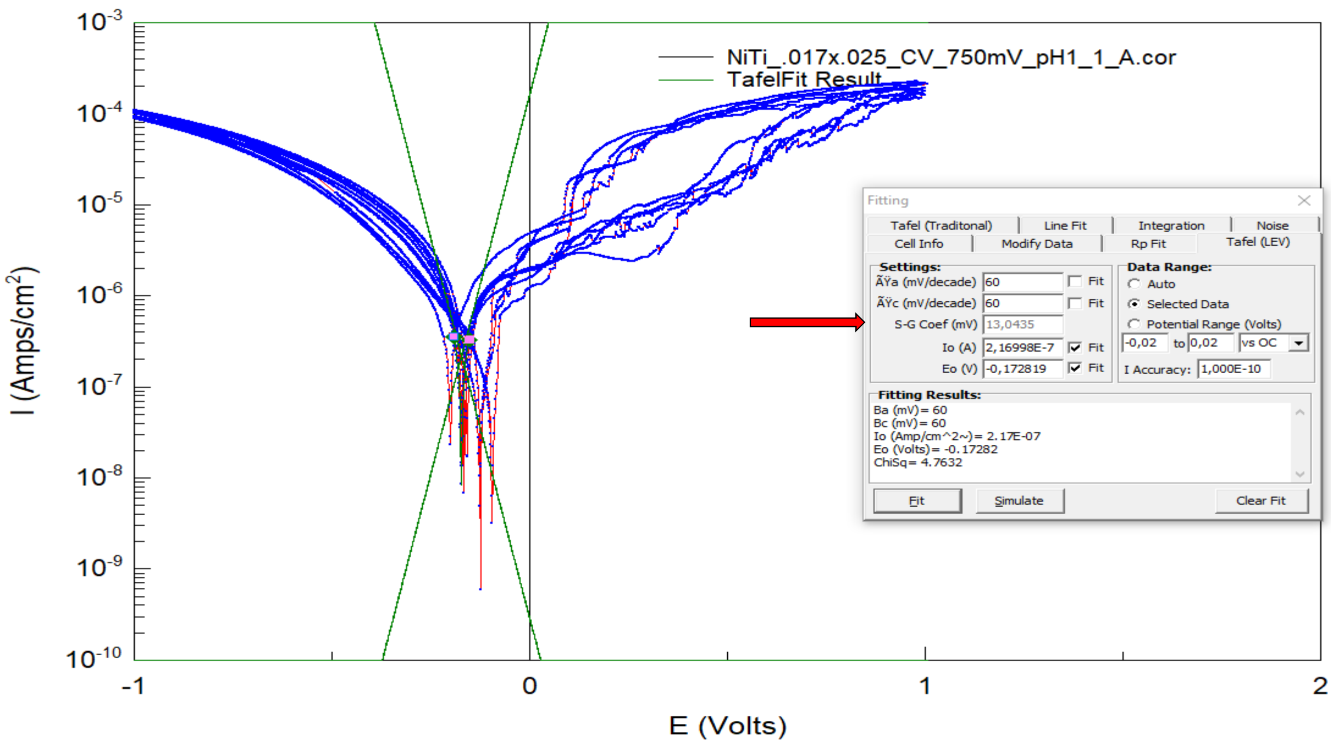

Figure S2. An example of the two-point is marked on the curve, and the ''Tafel LEV'' option is used to obtain the Stern coefficient.

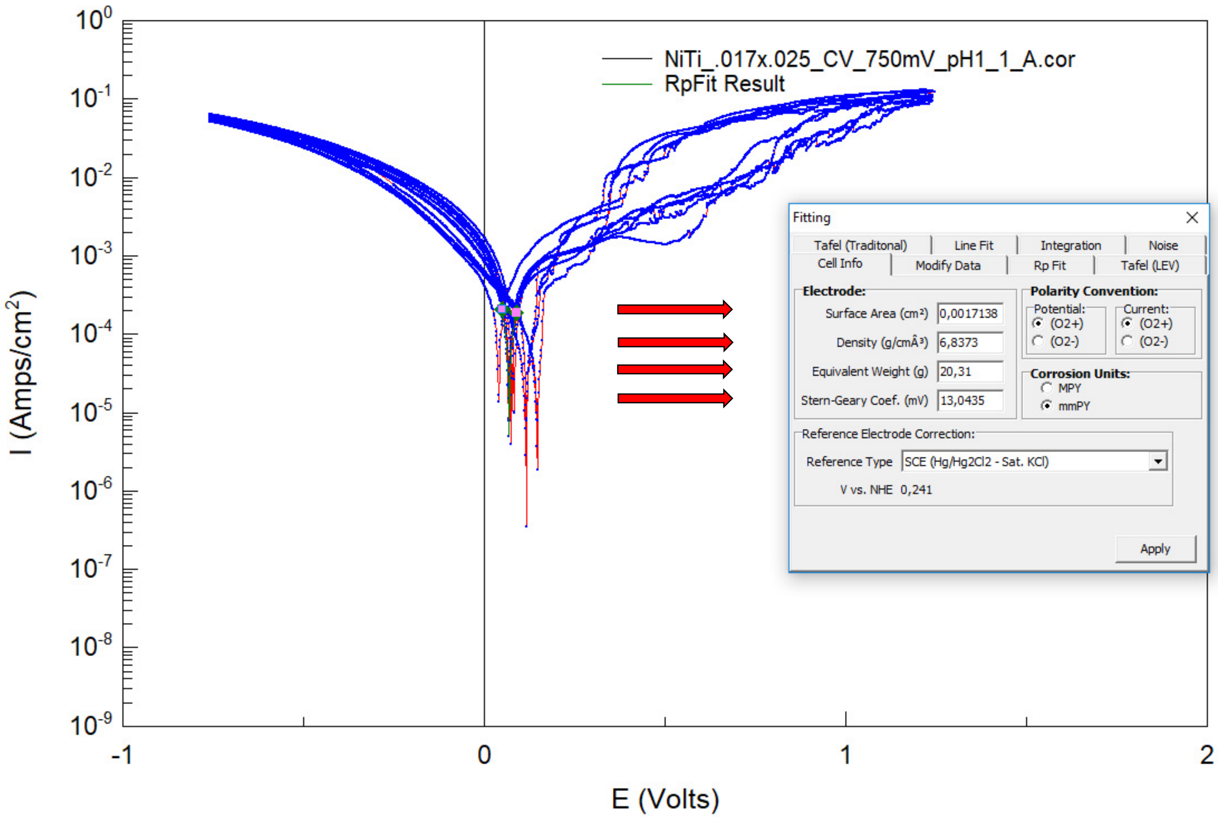

Figure S3. An example of cell information marked with red arrows.

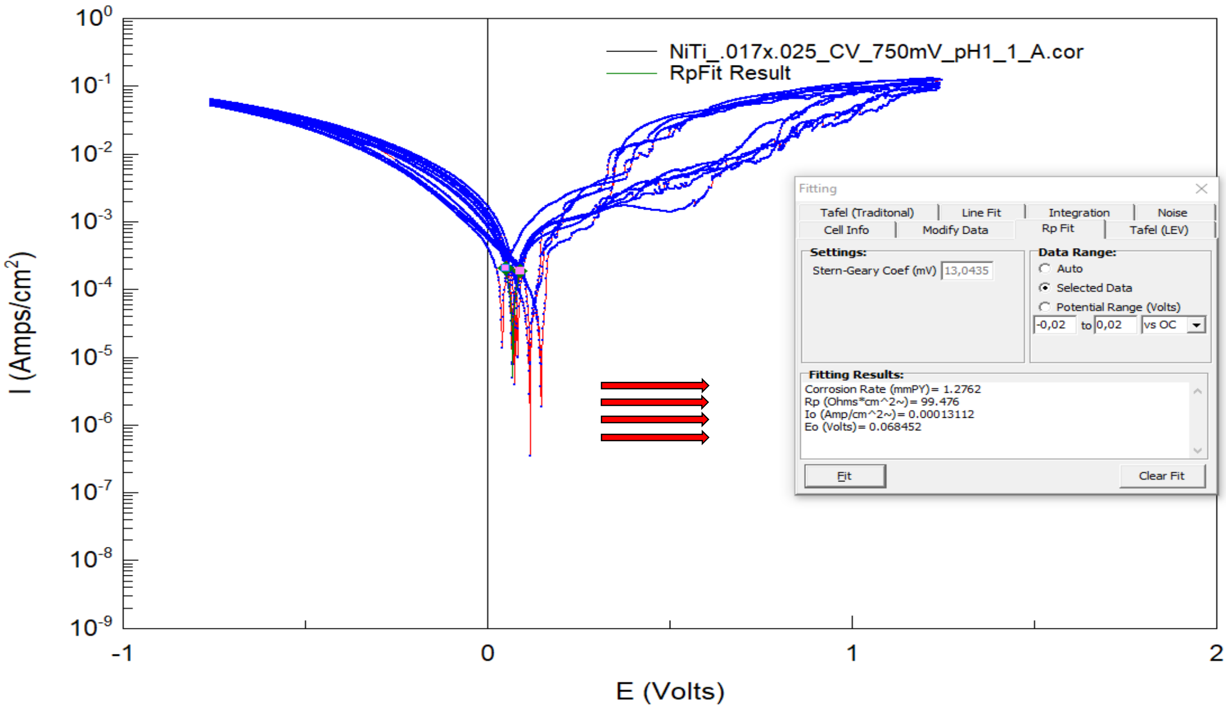

Figure S4. An example of the corrosion rate, polarization resistance (Rp), corrosion current density (I_0), and corrosion potential (E_0) calculation using ''RP – Fit''; the results are marked with red arrows.

Supplementary Materials B:

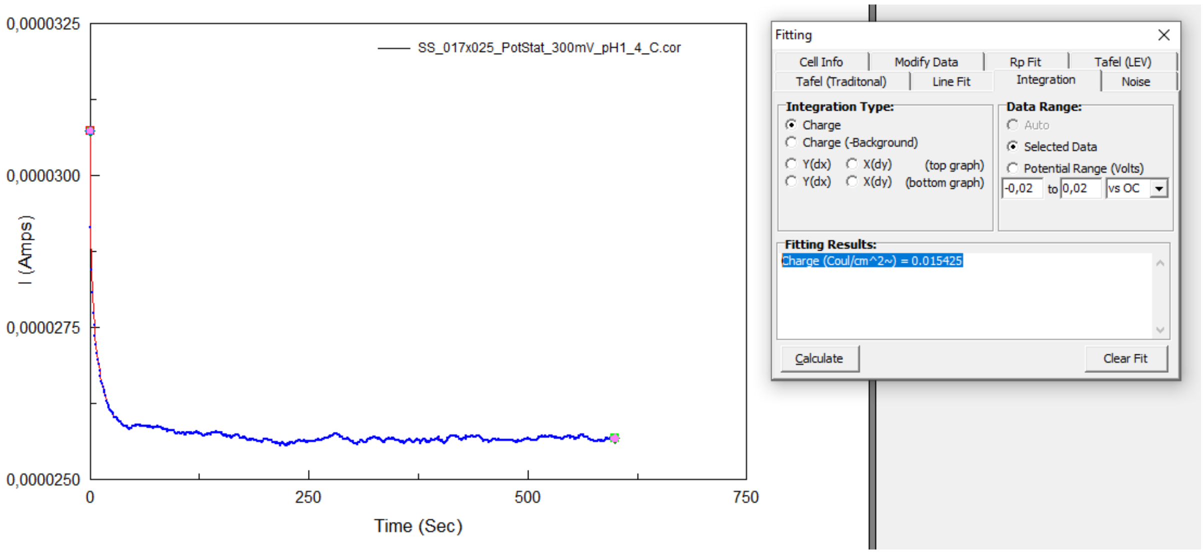

Figure S5. An example of the calculation of Q from I versus time curve using ''RP – Fit''.

Supplementary Materials C:

Table S1. Results of corrosion rate, E_0 from CV measurements and ICP-MS measurements from the NiTi_0.016″ × 0.022″.

														ICPMS RESULTS µg/L	
Orthodontic wire	Corr. Rate Cycle 1 (mmPY)	Eo (Volts)	Corr. Rate Cycle 2 (mmPY)	Eo (Volts)2	Corr. Rate Cycle 3 (mmPY)	Eo (Volts)3	Corr. Rate Cycle 4 (mmPY)	Eo (Volts)4	Corr. Rate Cycle 5 (mmPY)	Eo (Volts)5				Ti	Ni
NiTi_016x022_300mV_4_A	0,589	0,071	1,051	-0,118	1,610	0,143	1,939	-0,147	2,281	-0,150				138,190	193,360
NiTi_016x022_300mV_5_A	0,102	-0,031	0,675	-0,186	1,300	-0,171	2,003	-0,162	2,557	-0,149				75,690	140,970
NiTi_0,16x0,22_300mV_pH1_3_A	2,389	-0,185	3,865	-0,127	3,944	-0,126	4,185	-0,134	2,377	-0,044				no dat	no data
**Mean**	**1,027**	**-0,048**	**1,864**	**-0,144**	**2,285**	**-0,051**	**2,709**	**-0,148**	**2,405**	**-0,114**				**106,940**	**167,165**
**SD**	**1,205**	**0,129**	**1,743**	**0,037**	**1,445**	**0,170**	**1,279**	**0,014**	**0,140**	**0,061**				**44,194**	**37,045**
NiTi_0,16x0,22_750mV_pH1_1_A	2,169	-0,117	0,288	-0,043	2,484	-0,063	3,258	0,106	4,300	-0,113				no data	no data
NiTi_0,16x0,22_750mV_pH1_2_A	2,229	-0,178	4,245	-0,150	5,399	-0,153	5,297	-0,136	1,602	-0,113				''	''
NiTi_0,16x0,22_750mV_pH1_3_A	0,052	0,057	0,870	0,031	0,732	0,065	0,669	0,068	0,825	0,054				''	''
**MEAN**	**1,483**	**-0,079**	**1,801**	**-0,054**	**2,872**	**-0,050**	**3,075**	**0,013**	**2,242**	**-0,057**				''	''
**SD**	**1,240**	**0,122**	**2,136**	**0,091**	**2,358**	**0,110**	**2,319**	**0,130**	**1,824**	**0,096**				''	''
NiTi_016x022_300mV_1_B	0,082	0,044	1,162	-0,114	1,479	-0,119	0,656	-0,030	0,975	-0,045				7,610	<8
NiTi_016x022_300mV_2B	0,111	-0,068	0,619	-0,125	1,252	-0,125	2,066	-0,117	2,832	-0,113				29,670	46,680
NiTi_016x022_300mV_3B	0,279	-0,079	0,808	-0,115	1,445	-0,125	0,759	-0,059	0,887	-0,070				79,250	167,040
**MEAN**	**0,157**	**-0,034**	**0,863**	**-0,118**	**1,392**	**-0,123**	**1,160**	**-0,069**	**1,565**	**-0,076**				**38,843**	**106,860**
**SD**	**0,106**	**0,068**	**0,276**	**0,006**	**0,122**	**0,003**	**0,786**	**0,044**	**1,098**	**0,034**				**36,690**	**60,180**
NiTi_016x022_750mV_1_B	0,227	-0,073	0,635	-0,660	1,196	-0,128	2,110	-0,140	2,481	-0,145				118,220	249,530
NiTi_016x022_750mV_2B	0,110	-0,056	0,705	-0,125	1,451	-0,149	2,074	-0,150	1,876	-0,127				554,070	1046,330
NiTi_0166x022_750mV_pH1_3B	0,157	-0,073	1,001	-0,098	1,741	-0,141	1,976	-0,131	0,624	-0,057				1639,390	3128,770
**MEAN**	**0,165**	**-0,067**	**0,780**	**-0,294**	**1,463**	**-0,139**	**2,053**	**-0,140**	**1,660**	**-0,110**				**770,560**	**1474,877**
**SD**	**0,059**	**0,010**	**0,194**	**0,317**	**0,273**	**0,011**	**0,069**	**0,010**	**0,947**	**0,046**				**783,352**	**1486,689**
NiTi_016x022_750mV_1_C	0,206	-0,072	0,554	-0,073	1,150	-0,134	2,219	-0,151	1,771	-0,116				397,150	533,890
NiTi_016x022_750mV_2_C	0,115	-0,040	0,572	-0,123	0,186	-0,030	0,095	0,018	0,819	-0,109				422,550	665,620
NiTi_016x022_750mV_3_C	0,108	-0,048	0,671	-0,120	1,286	-0,143	1,972	-0,152	1,845	-0,124				508,460	882,780
**MEAN**	**0,143**	**-0,053**	**0,599**	**-0,105**	**0,874**	**-0,102**	**1,429**	**-0,095**	**1,478**	**-0,116**				**442,720**	**694,097**
**SD**	**0,055**	**0,017**	**0,063**	**0,028**	**0,600**	**0,063**	**1,162**	**0,098**	**0,572**	**0,008**				**58,332**	**176,180**
NiTi_016x022_300mV_1_C	0,020	0,023	0,278	-0,104	0,224	-0,043	0,520	-0,116	2,089	-0,145				17,520	8,160
NiTi_016x022_300mV_2_C	0,019	0,025	0,123	0,006	0,162	-0,035	0,739	-0,075	0,987	-0,086				4,930	6,300
NiTi_016x022_300mV_3_C	0,023	0,021	0,838	-0,118	1,165	-0,160	1,413	-0,131	0,308	-0,039				17,890	22,130
**MEAN**	**0,021**	**0,023**	**0,413**	**-0,072**	**0,517**	**-0,079**	**0,891**	**-0,107**	**1,128**	**-0,090**				**13,447**	**12,197**
**SD**	**0,002**	**0,002**	**0,376**	**0,068**	**0,562**	**0,070**	**0,465**	**0,029**	**0,899**	**0,053**				**7,378**	**8,653**
